# Supplementary figures and images for: Cell cycle transcriptomics of Capsaspora provides insights into the evolution of cyclin-CDK machinery
Source: PLoS Genet. 2020 Mar 16;16(3):e1008584. doi: 10.1371/journal.pgen.1008584 (PMC7098662; doi:10.1371/journal.pgen.1008584)

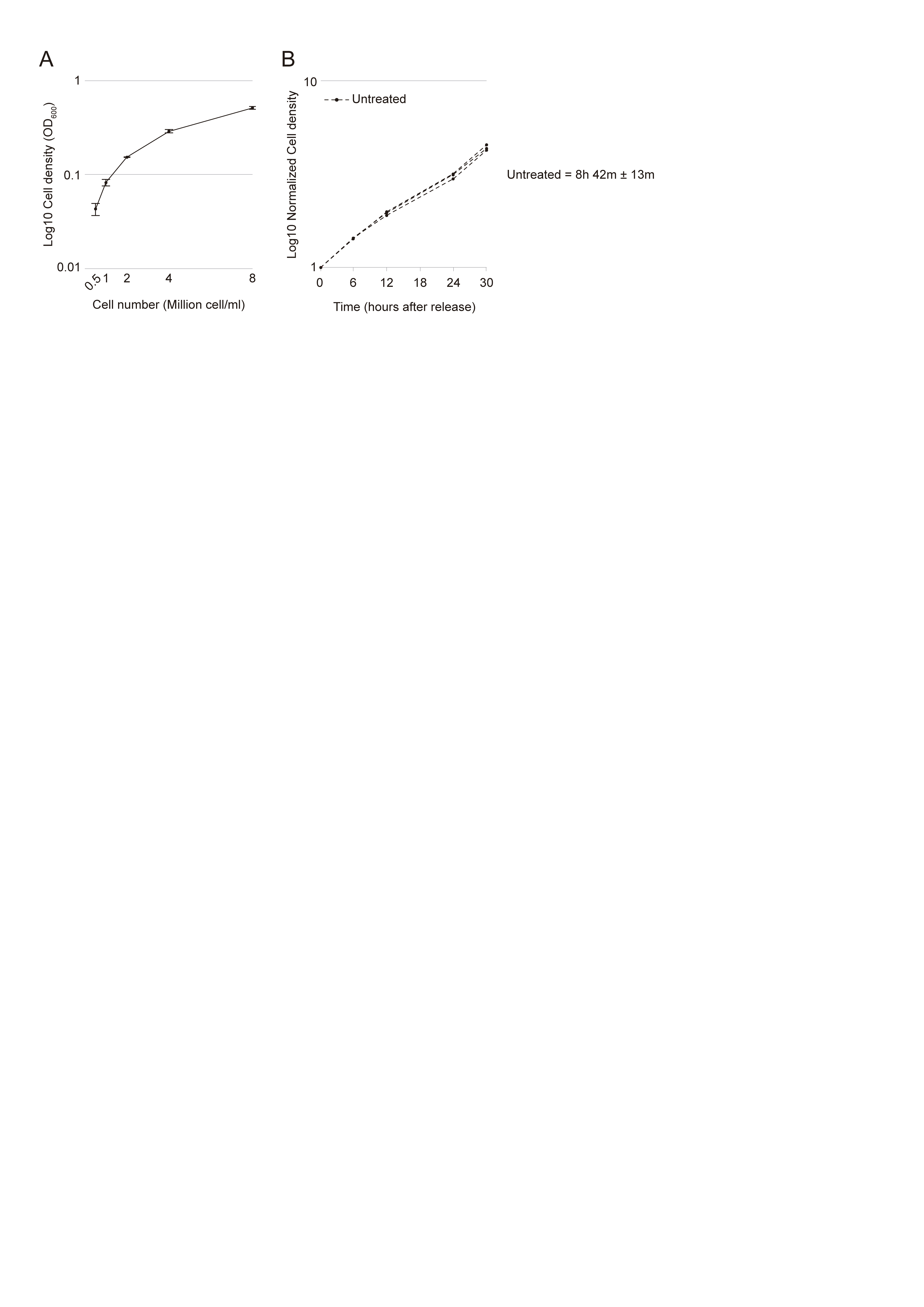

Supplement: S1 Fig — A: Cell density curve (optical density, OD = 600 nm) at different cell concentrations of Capsaspora (three replicates per condition). Values of cell concentration around OD600 = 0.1 were used to seed cultures and calculate the doubling time of Capsaspora in B. B: Growth curve (logarithmic scale) of non-synchronized Capsaspora cell cultures (three replicates). Doubling time is calculated from three independent replicates. (TIF) [file pgen.1008584.s001.tif]

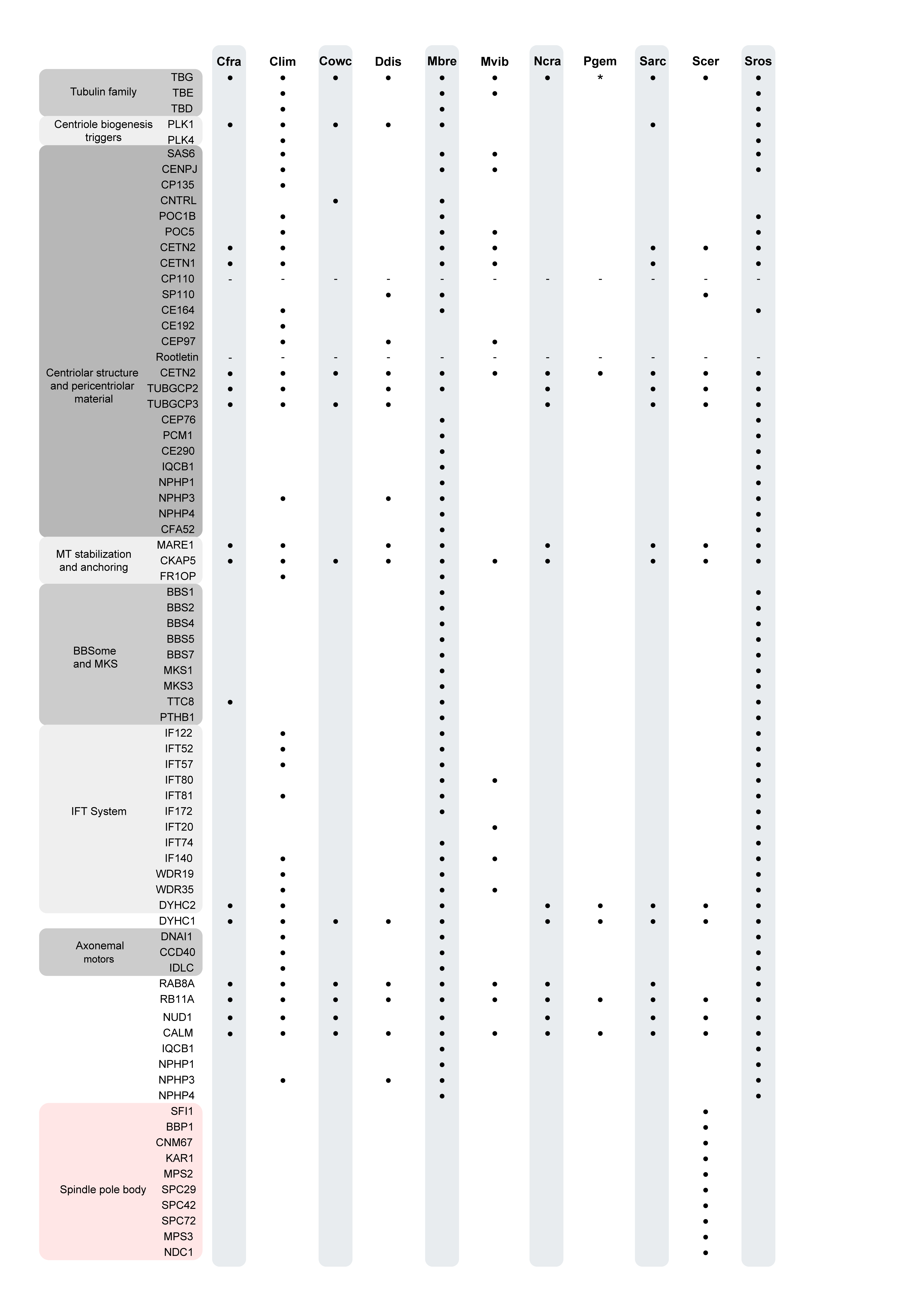

Supplement: S2 Fig — Presence/absence matrix of the animal and yeast microtubule organizing proteins across Salpingoeca rosetta (Sros), Monosiga brevicolis (Mbre), Ministeria vibrans (Mvib), Capsaspora owczarzaki (Cowc), Creolimax fragrantissima (Cfra), Sphaeroforma arctica (Sarc), Corallochytrium limacisporum (Clim), Saccharomyces cerevisiae (Scer), Neurospora crassa (Ncra), and Dictyostelium discoideum (Ddis). List of proteins from Carvalho-Santos et al., 2011, Hodges et al., 2010, and Uniprot. Classification adapted from Carvalho-Santos et al., 2011, and Hodges et al., 2010. (TIF) [file pgen.1008584.s002.tif]

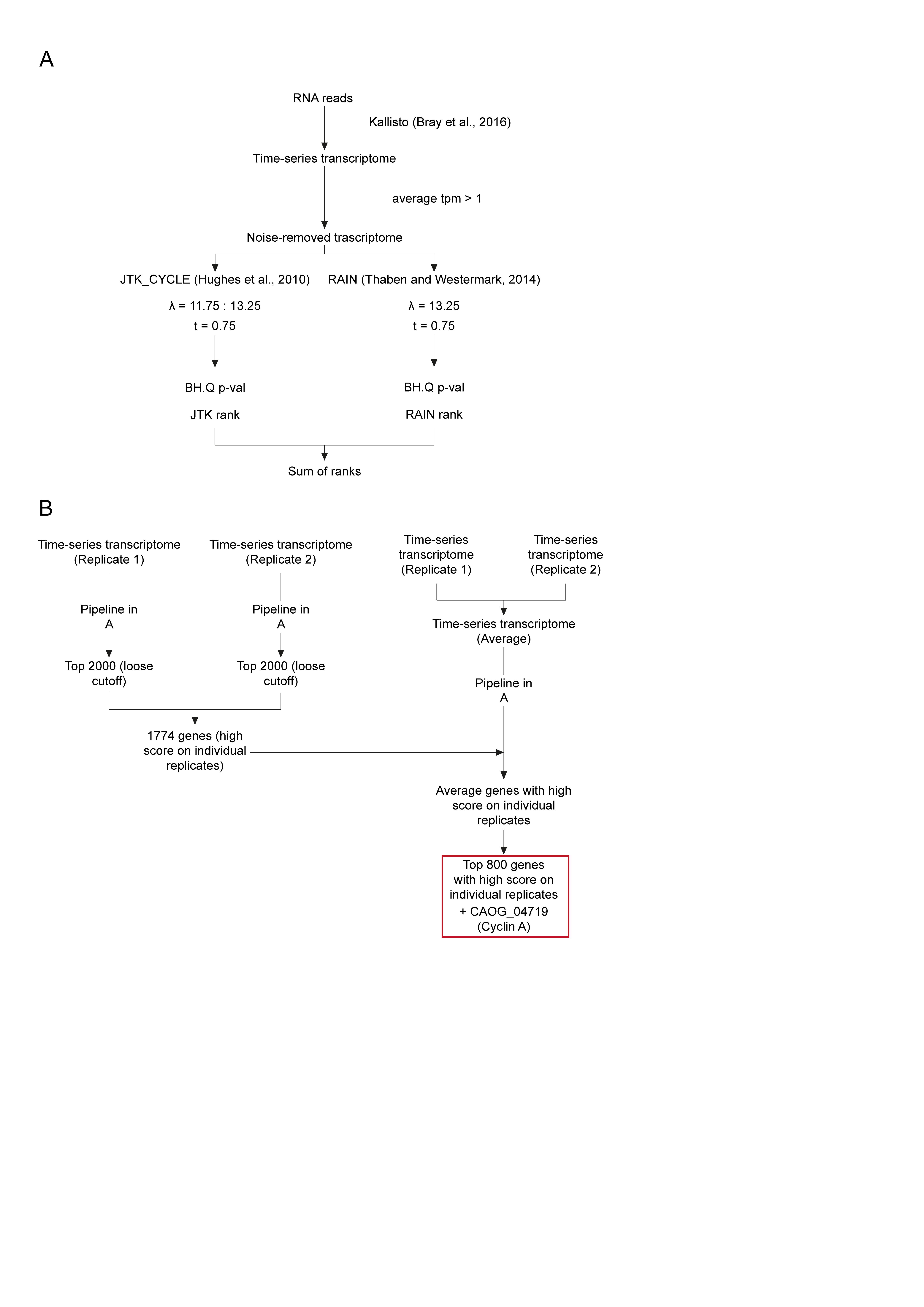

Supplement: S3 Fig — A: Pipeline for ranking the transcripts on each experiment. RNA reads were processed using Kallisto, noise transcripts were filtered out, and JTK and RAIN were run in parallel with the indicated setup. Bonferroni-corrected p-values were ranked, and the sum of ranks was used as a final rank. B: Pipeline used to detect periodic transcripts in Capsaspora. Samples were treated separately to detect periodic transcripts with a loose cutoff. This gene set was used to filter periodic genes ranked from an average dataset of the two replicates, out of which the top 800 (10% of the number of genes in Capsaspora) were selected. (TIF) [file pgen.1008584.s003.tif]

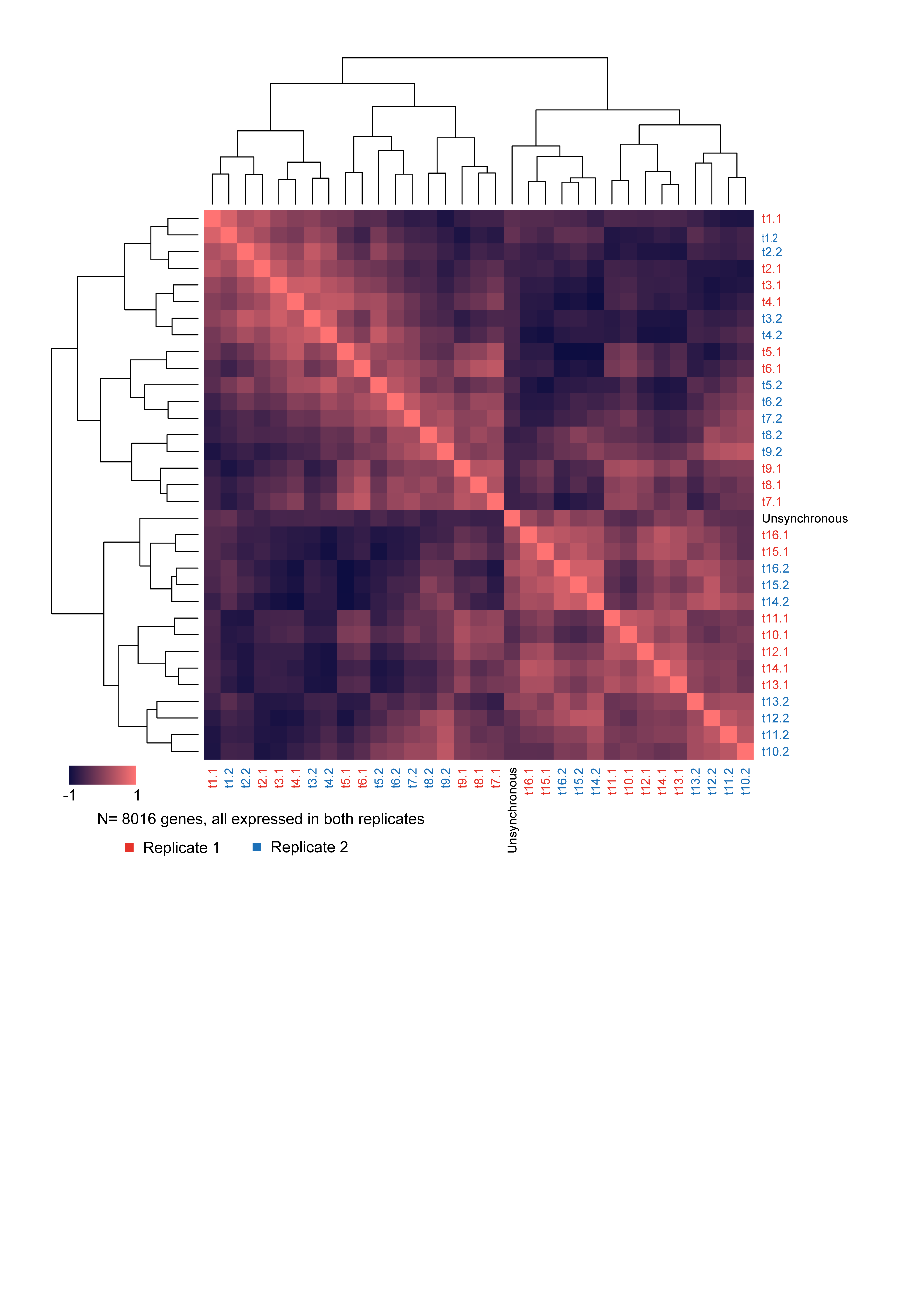

Supplement: S4 Fig — All the genes with an average expression level above 1 tpm in both replicates were used. (TIF) [file pgen.1008584.s004.tif]

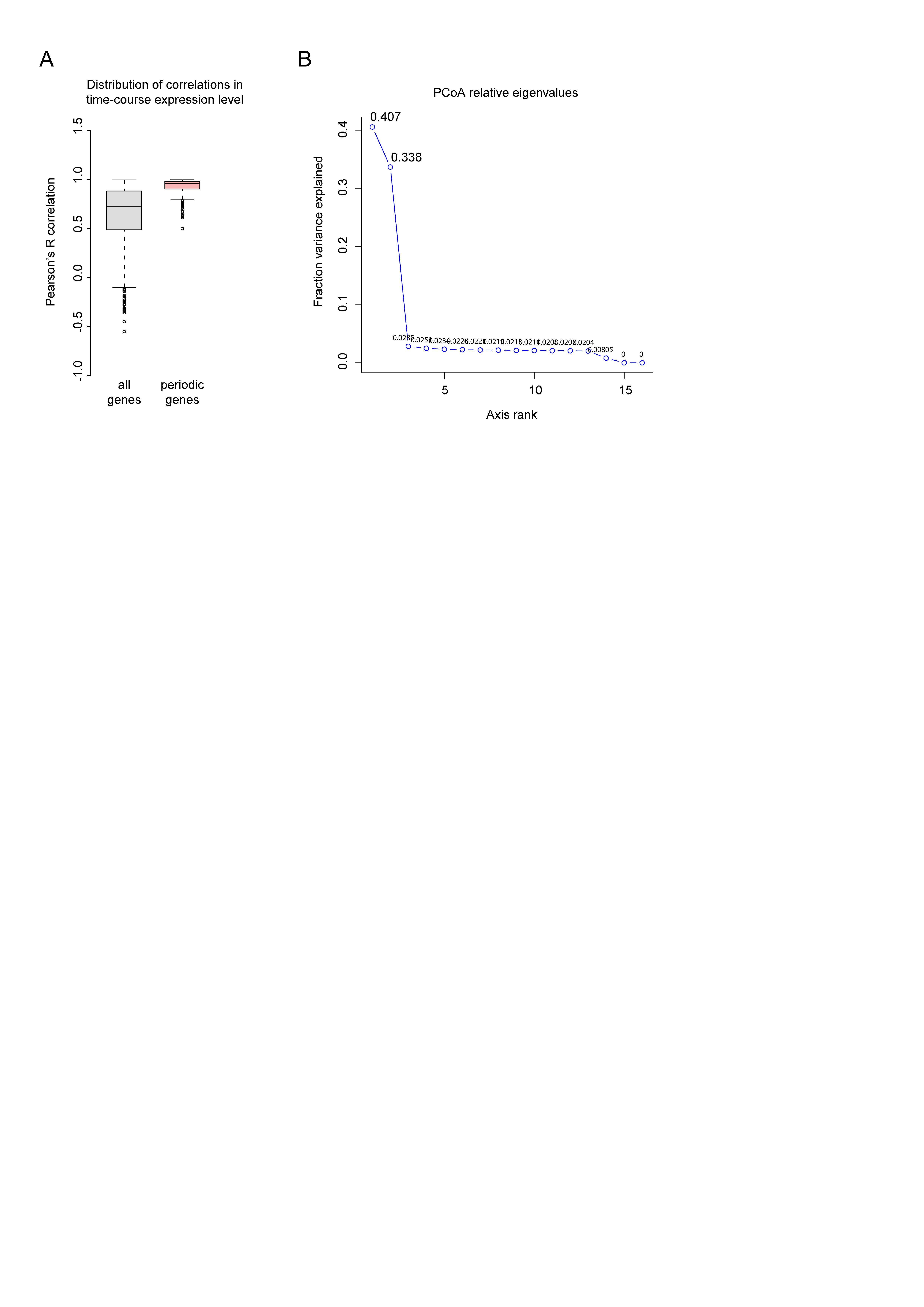

Supplement: S5 Fig — A: Distributions of Pearson correlation between replicates of a set of randomly chosen 801 genes and the 801 genes defined as periodic. B: Fraction of variance explained by the different relative eigenvalues of the principal coordinate analysis (see Fig 3D). (TIF) [file pgen.1008584.s005.tif]

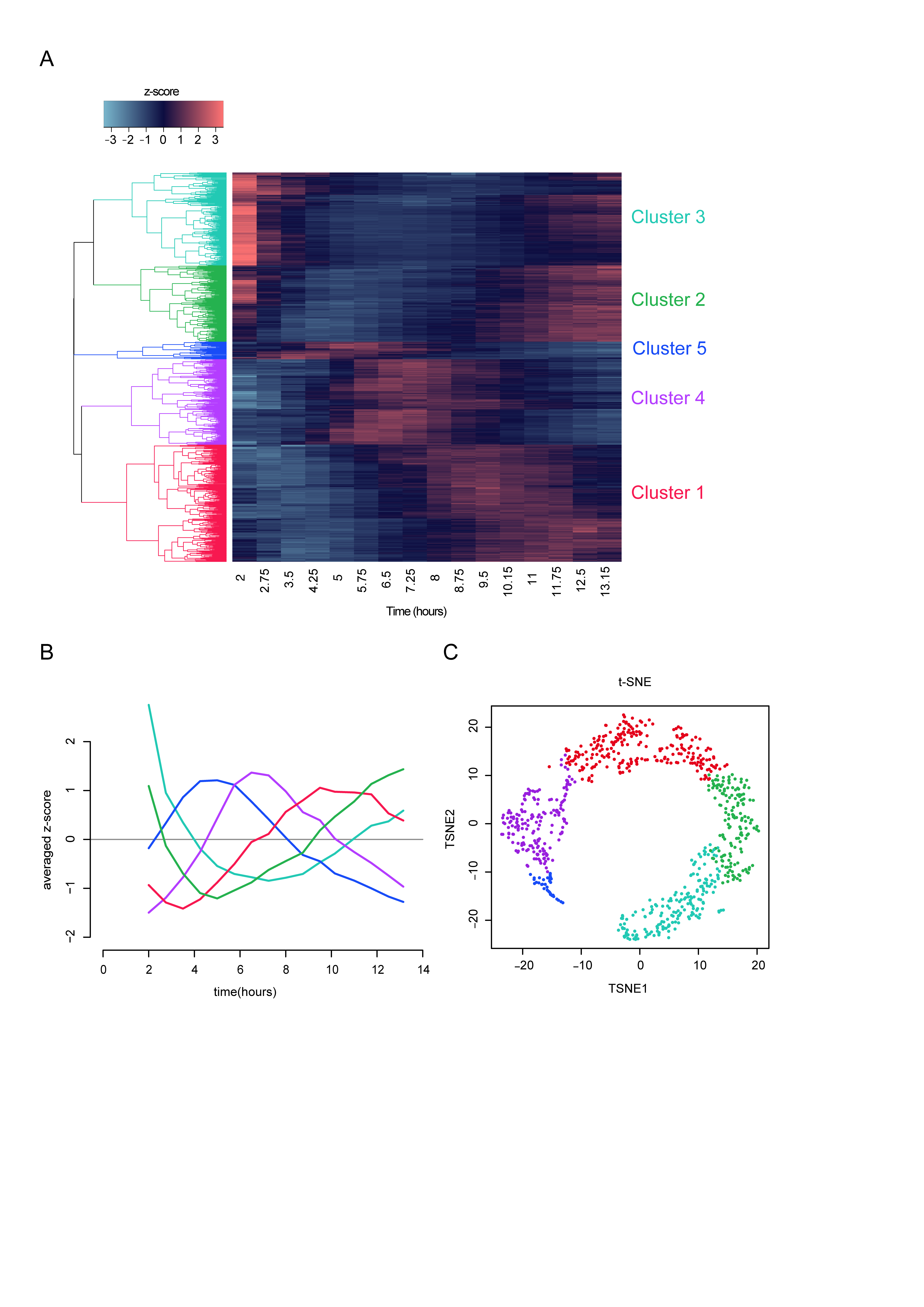

Supplement: S6 Fig — A: Hierarchical clustering of the expression profiles of the 801 periodic genes found in Capsaspora, and the color equivalences with the final clusters shown at Fig 4A. B: Average expression level of Capsaspora periodic genes grouped by hierarchical clustering. C: t-SNE plot of all 801 genes in the periodic transcriptional program of Capsaspora, showing circle pattern as in Fig 3D. Color code in both figures follows the color code in Fig 4. (TIF) [file pgen.1008584.s006.tif]

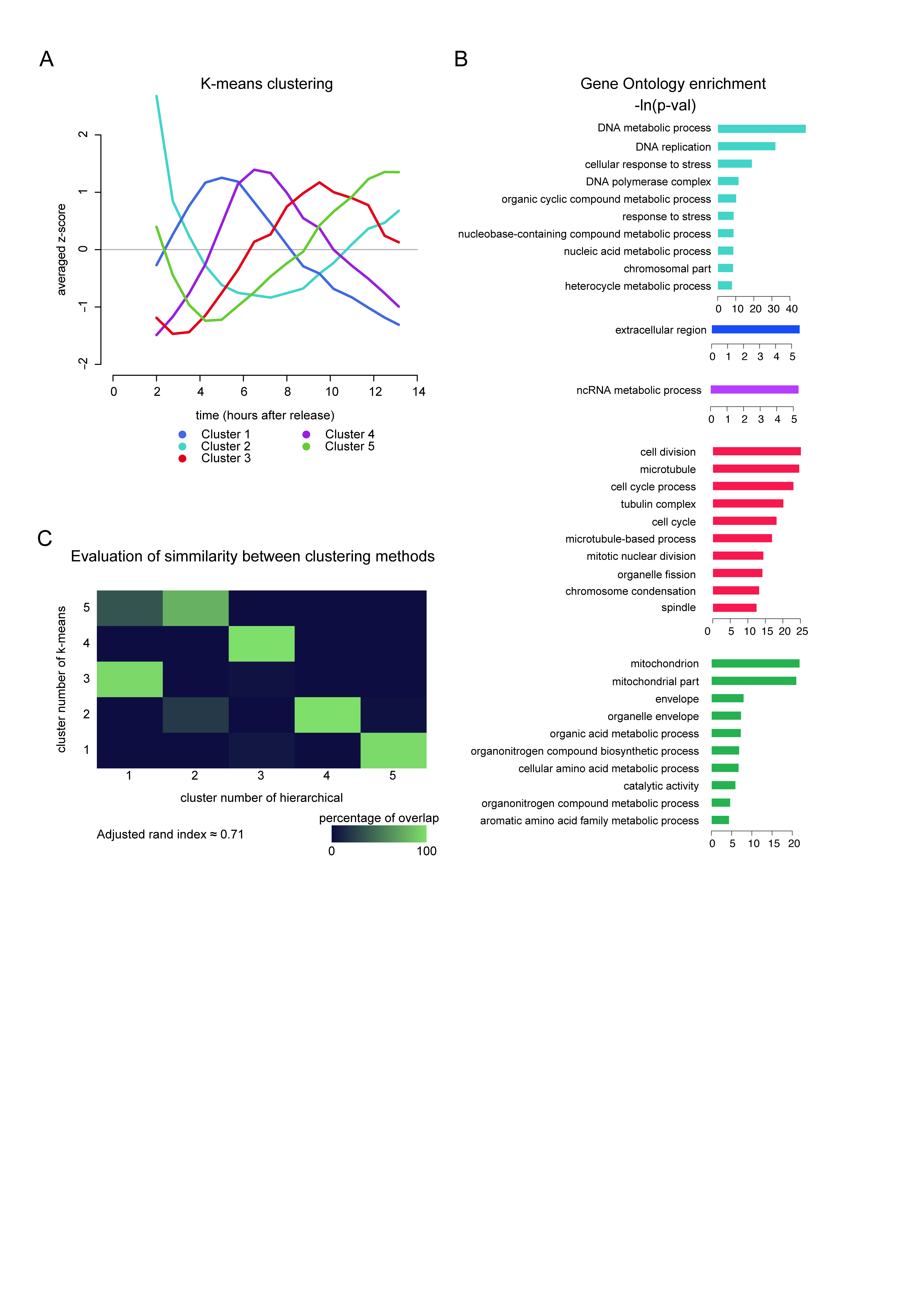

Supplement: S7 Fig — A: Average expression level of Capsaspora periodic genes grouped by k-means clustering. B: Top ten enriched GO terms of each cluster of periodic genes generated by k-means clustering. GO terms were considered significant when Bonferroni-corrected p-value was lower than 0.05. Full list available at S3 Fig. C: Agreement between clustering methods. Heatmap showing the percentage of overlap between clusters by two methods. Overlap is calculated as the number of genes belonging to the same pair of clusters divided by the size of the smallest cluster in the pair. (TIF) [file pgen.1008584.s007.tif]

# S8 Fig

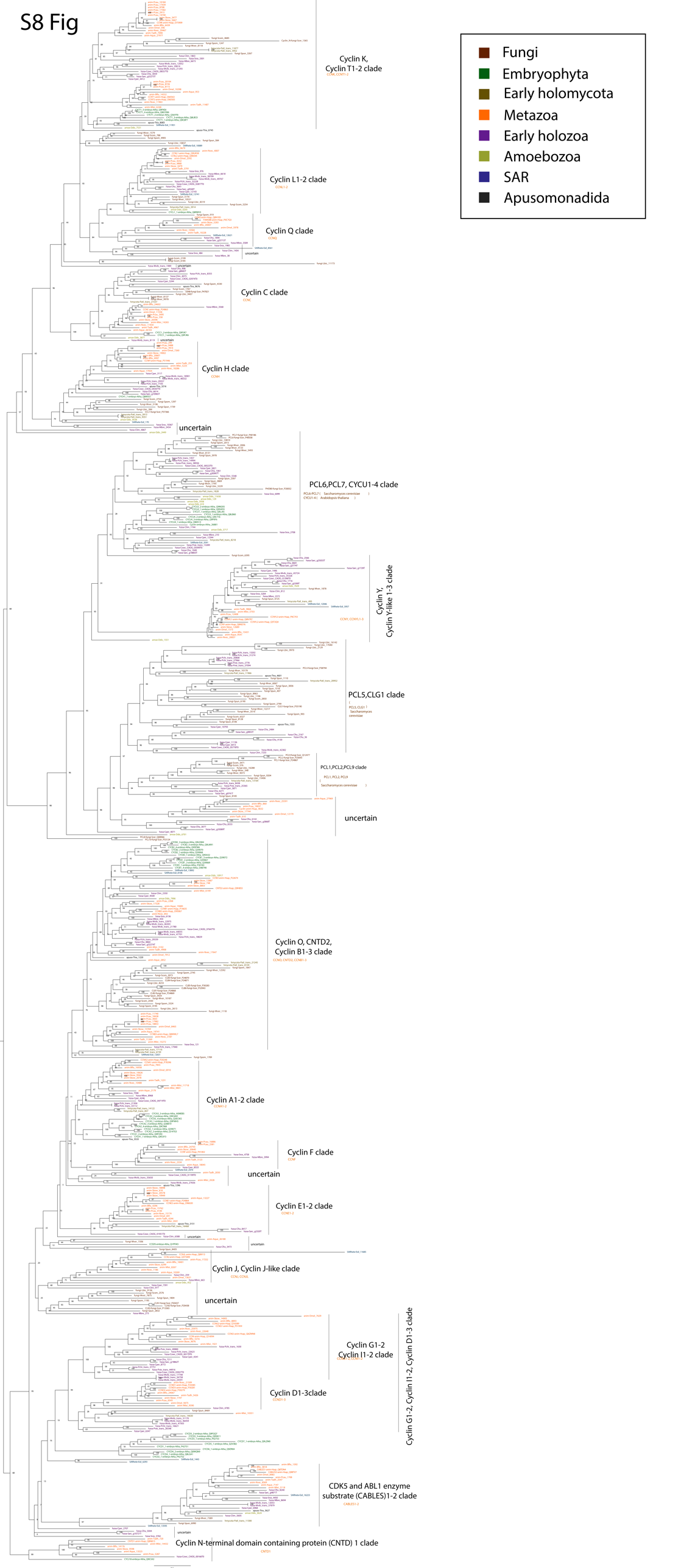

Supplement: S8 Fig — Nodal support values (1000- bootstrap replicates by UFBoot) are shown in all nodes. Eukaryotic sequence names are abbreviated with the four-letter code (see Methods) and colored according to their major taxonomic group (see panel). (PDF) [file pgen.1008584.s008.pdf]

S9 Fig

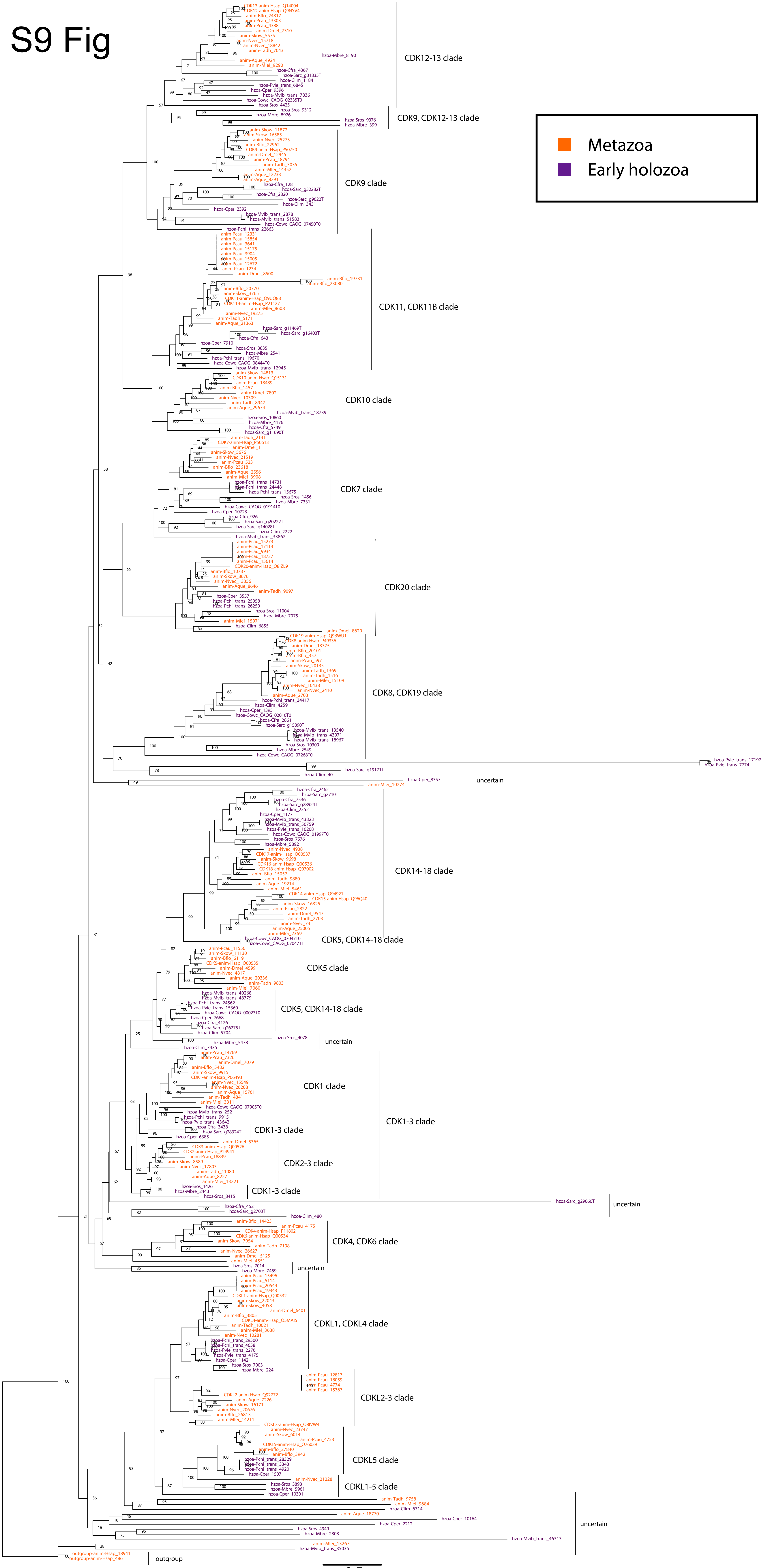

Supplement: S9 Fig — Statistical support values (1000-replicates UFBoot) are shown in all nodes. Eukaryotic sequence names are abbreviated with the four-letter code (see Methods) and colored according to their major taxonomic group (see panel). (PDF) [file pgen.1008584.s009.pdf]

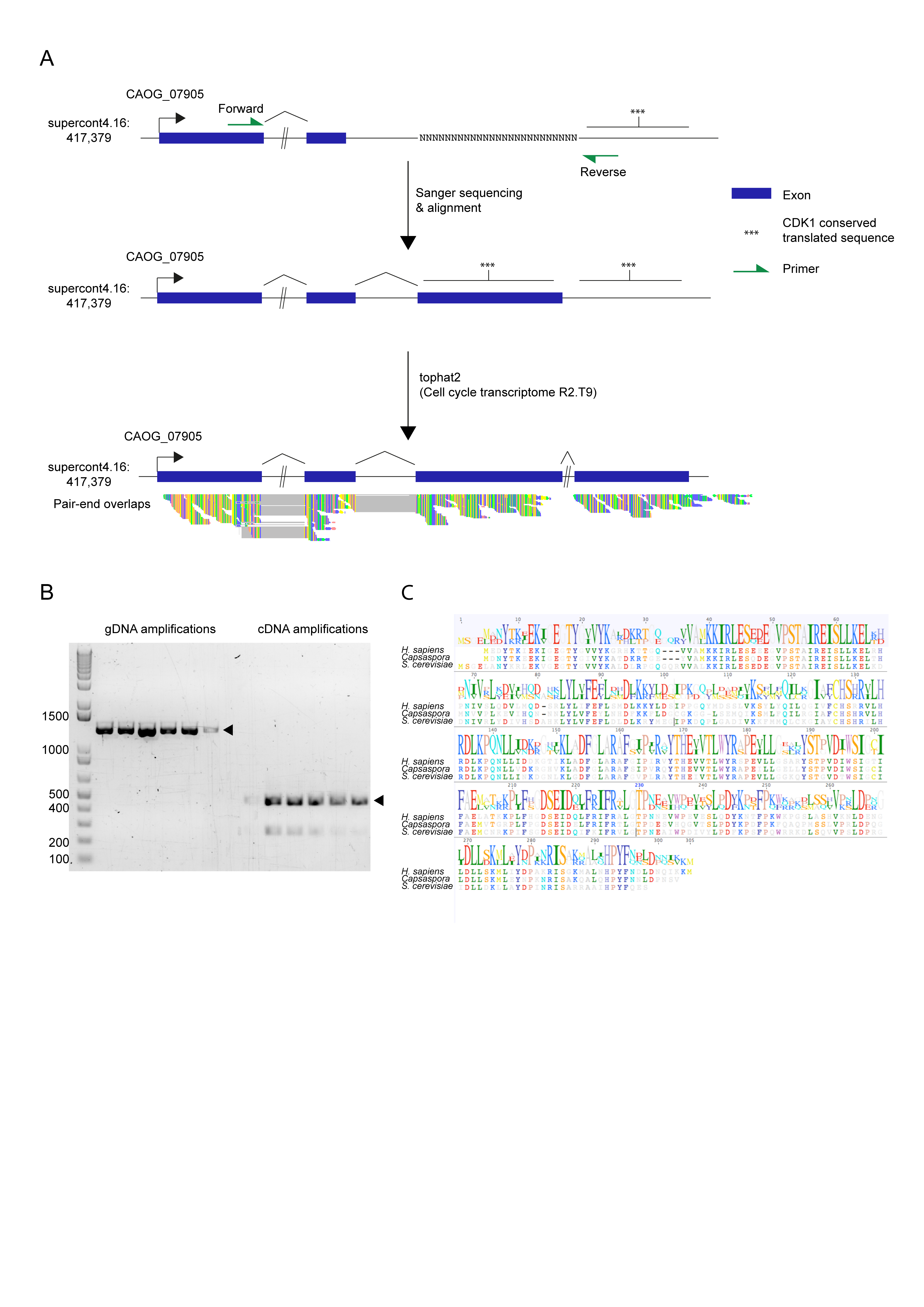

Supplement: S10 Fig — A: Schematic representation of the genomic locus of Capsaspora CDK1-3 gene showing exons, splicing sites, non-annotated regions of predicted sequence, and mapping of mRNA reads. B: PCR amplifications of Capsaspora CDK1 using primers detailed in Methods and A, using genomic DNA and cDNA as templates. Arrows indicate size of the products sent for sequencing. C: Alignment of H. sapiens and S. cerevisiae CDK1 genes, and the Capsaspora updated CDK1-3 sequence, using Geneious v8.1.9. (TIF) [file pgen.1008584.s010.tif]

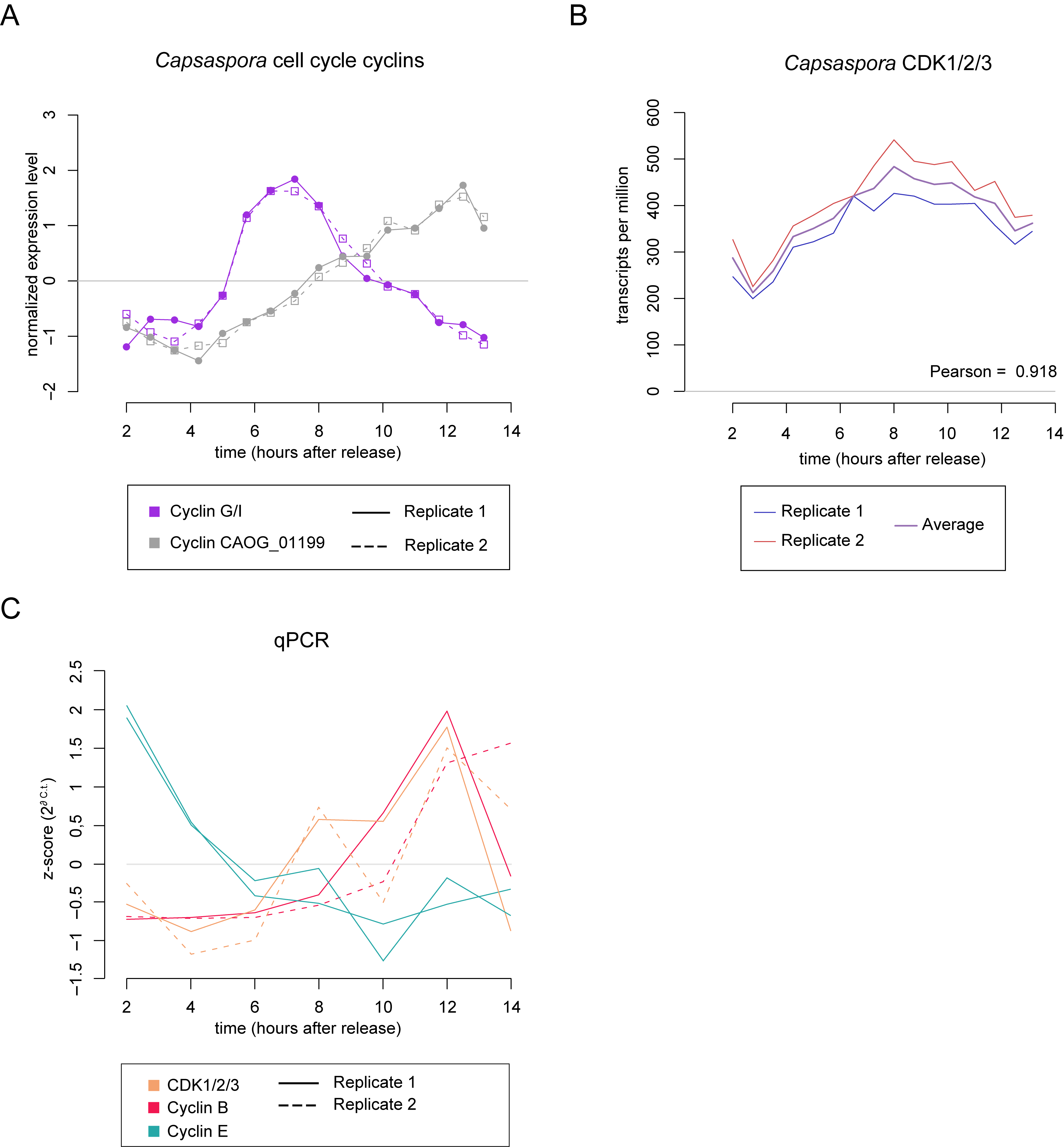

Supplement: S11 Fig — A: Gene expression profile of cyclin G/I and an unidentified cyclin gene (CAOG_01199) found in Capsaspora. B: Total amount of Capsaspora CDK1/2/3 throughout the cell cycle. C: Dynamics of the cyclin-CDK system using real-time PCR. Normalized gene expression profiles of cyclins B, E, and CDK1/2/3 in two independent biological replicates of synchronized Capsaspora cultures. (TIF) [file pgen.1008584.s011.tif]

# S12 Fig

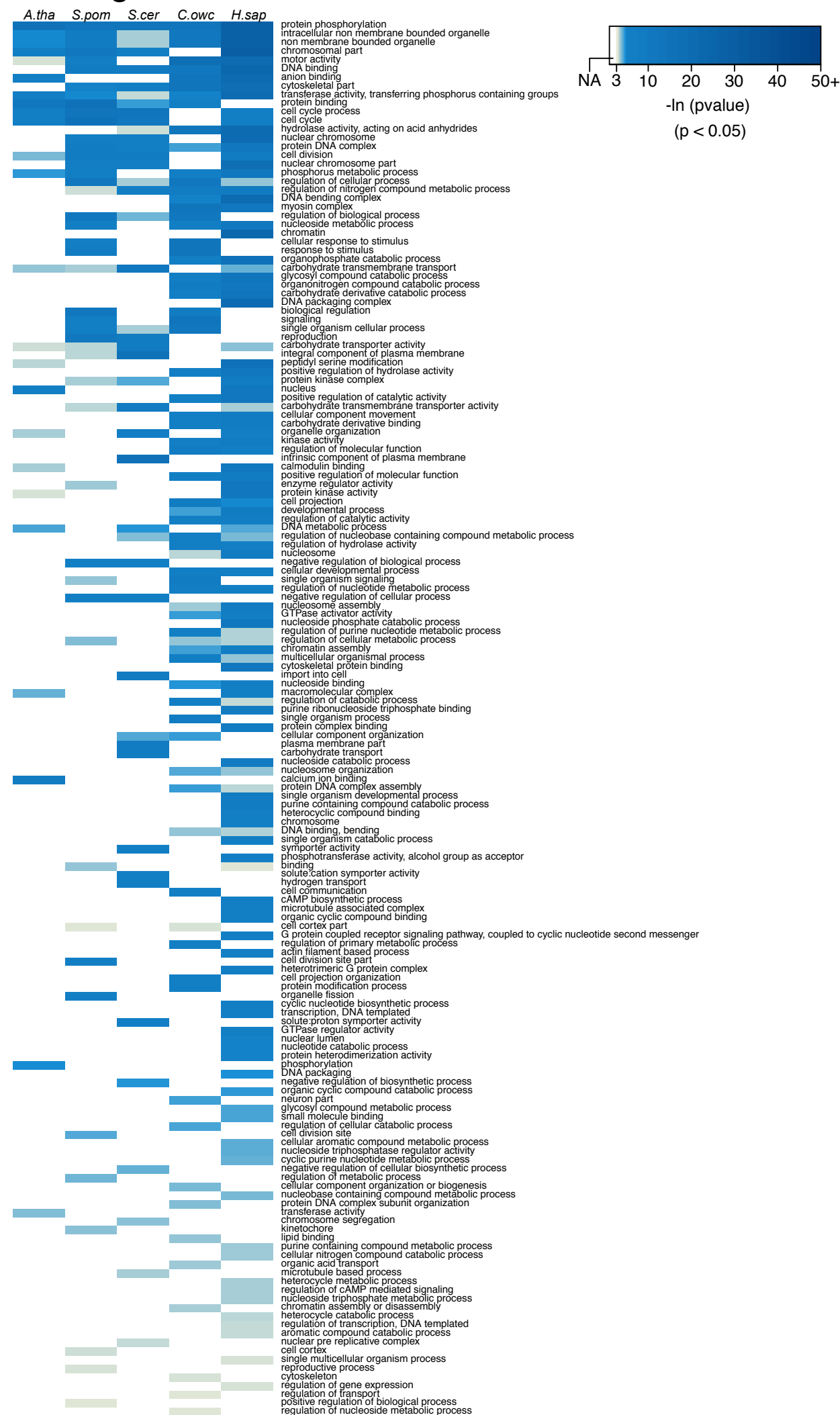

Supplement: S12 Fig — (PDF) [file pgen.1008584.s012.pdf]

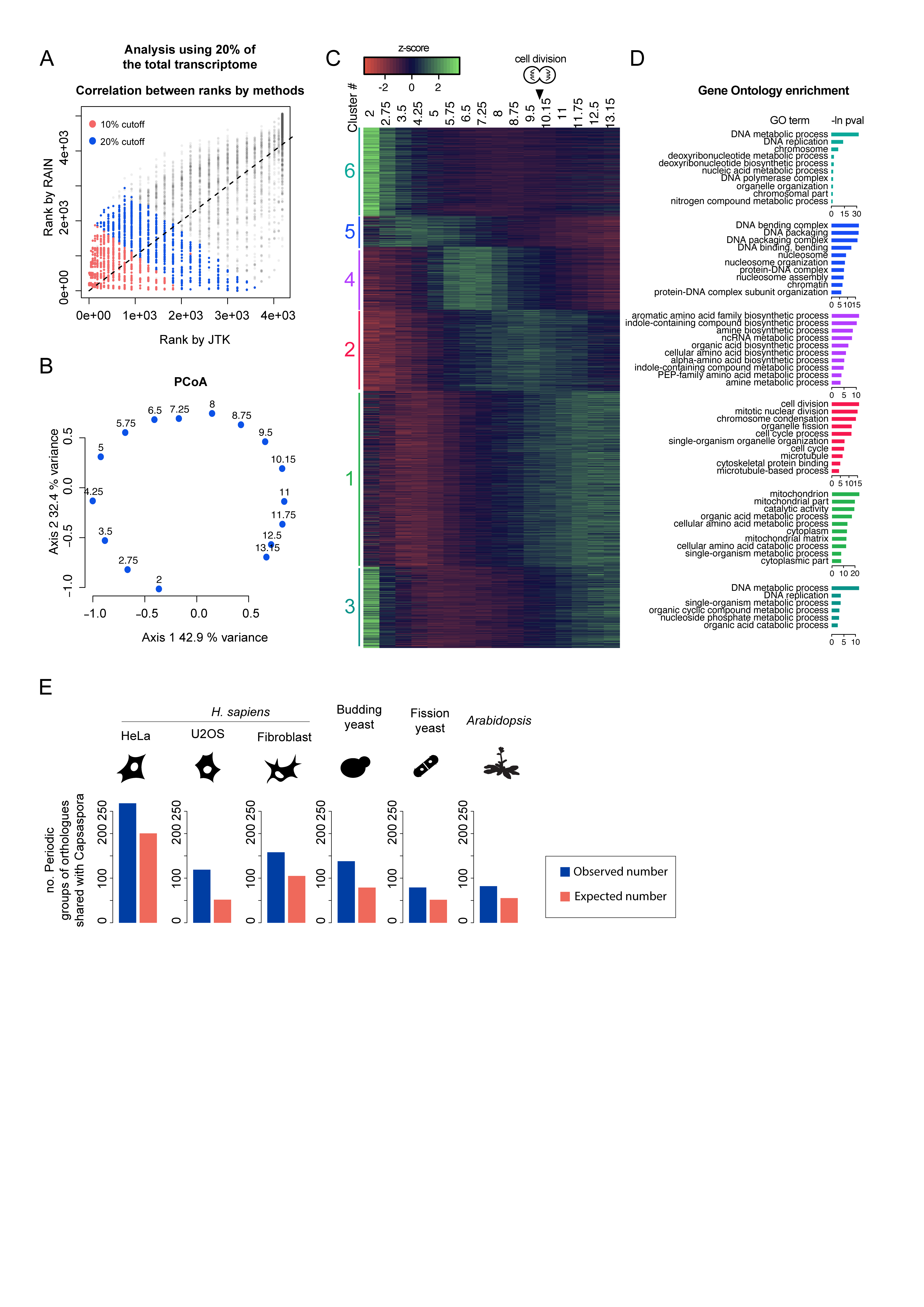

Supplement: S13 Fig — A: Scatter plot replicating Fig 3B, showing in blue all the genes additionally taken into account. Colored dots represent the 1600 genes that were finally taken as periodic in this reanalysis. B: Principal coordinate analysis using the dataset of 1600 genes. C: heatmap of gene expression level depicting six clusters detected by Euclidean distance hierarchical clustering. Clusters were rearranged to visually represent their expression peaks over time. Black arrow and dividing cell indicate time of cell division (see Fig 2). D: Gene ontology enrichment analysis of the six clusters represented in C. E: Bar plots indicating the amount of shared periodic orthogroups and/or periodic one-to-one orthologues between pairs of cell types or species, using the dataset of 1600 periodic genes in Capsaspora. (TIF) [file pgen.1008584.s013.tif]

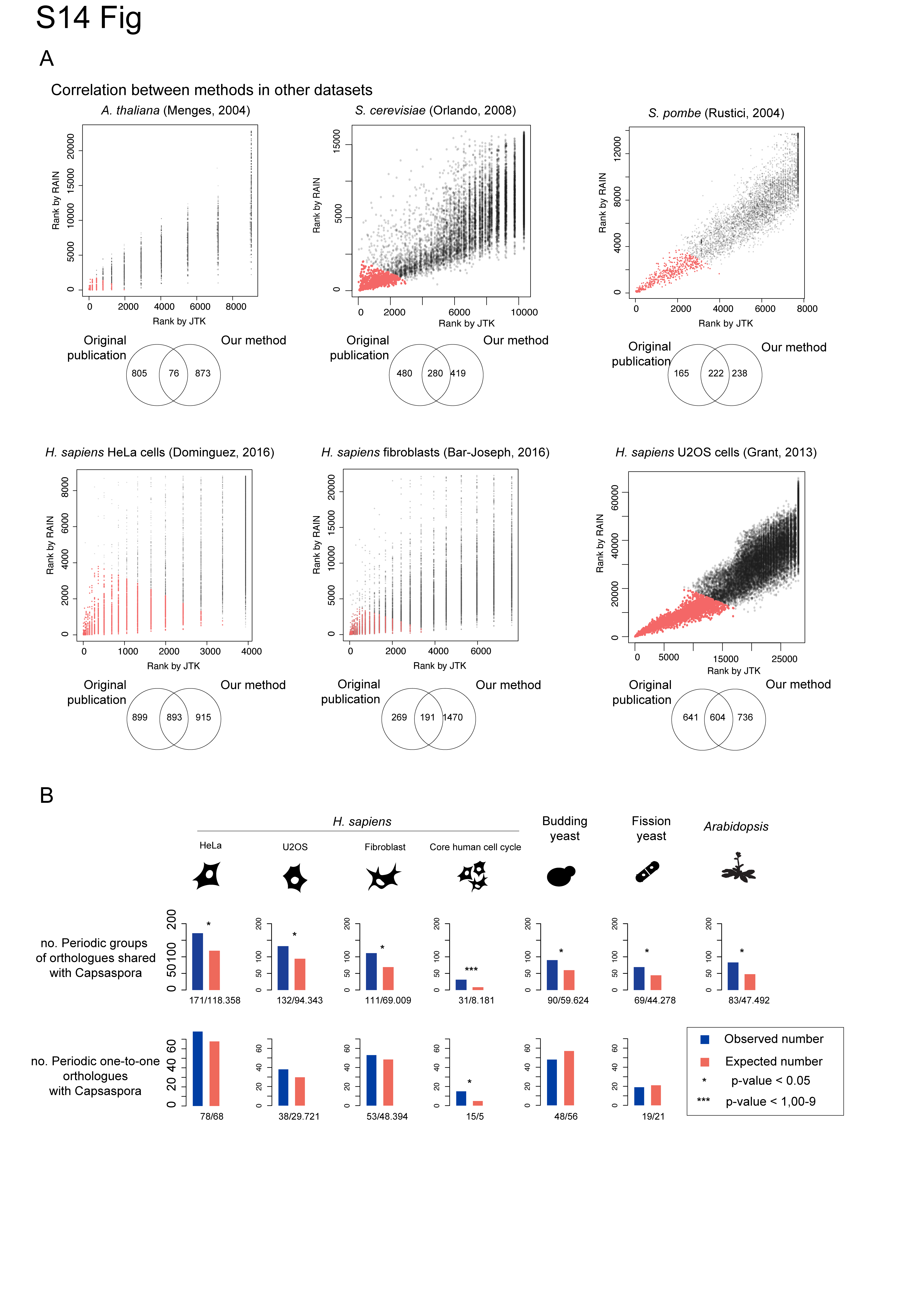

Supplement: S14 Fig — A: Scatter plots of ranks by JTK and RAIN for each dataset of each species used in the comparative analysis (see Fig 6D and Results section). Datasets were processed as indicated in S15 Text, and Material and methods. Depending on the dataset, we could recover between one third and more than half of the originally described periodic genes, except Arabidopsis where the agreement was very low. B: Bar plots indicating the amount of shared periodic orthogroups and/or periodic one-to-one orthologues between pairs of cell types or species, using our own lists of periodic genes. P-values of all the binomial tests are provided in S17 Text. (TIF) [file pgen.1008584.s014.tif]

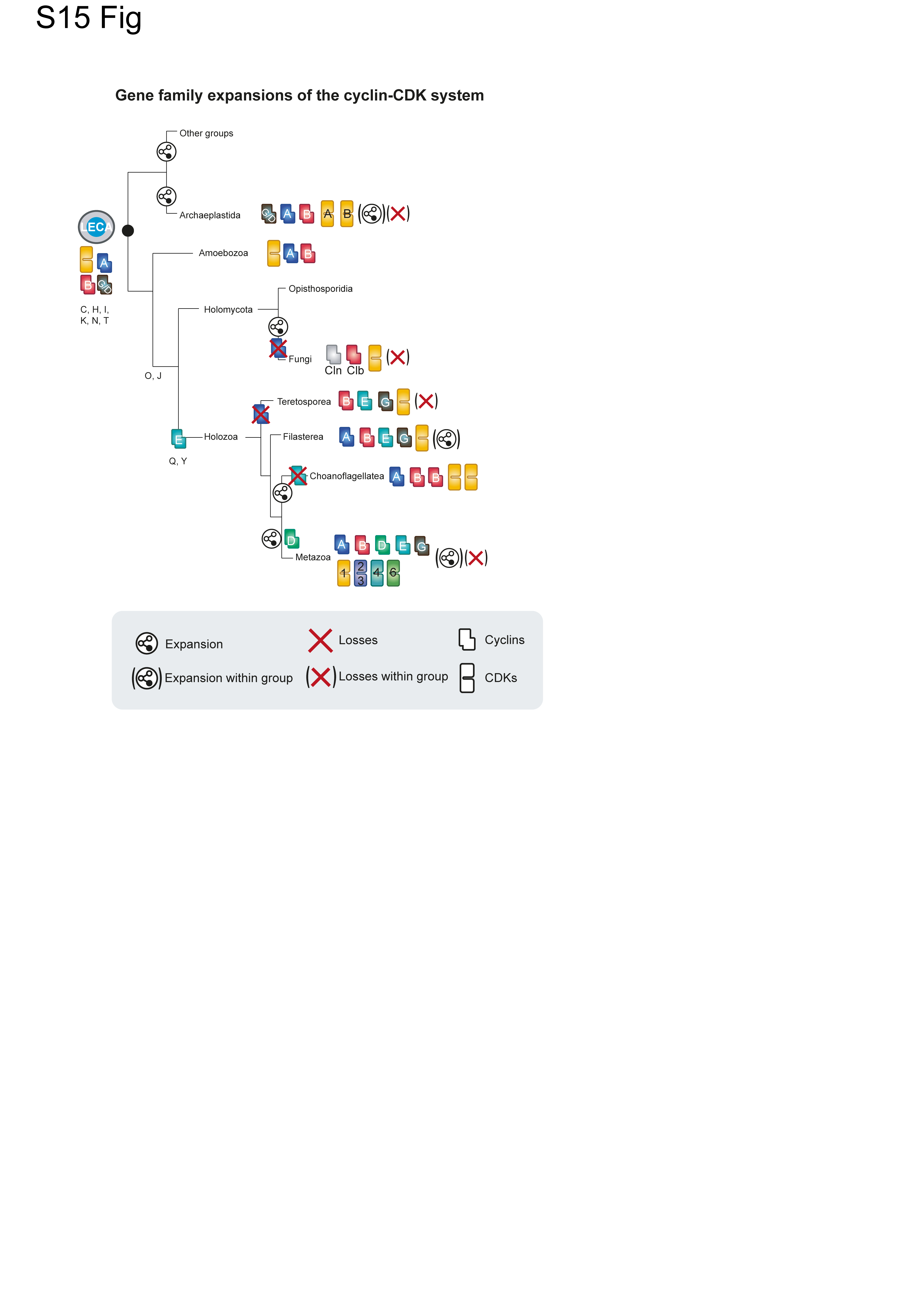

Supplement: S15 Fig — Phylogenetic tree of eukaryotes displaying the different expansions, gains and losses of the main cyclin and CDK subfamilies with a known role in cell cycle regulation, in different eukaryotic groups. Events within groups refers to some species within the group showing additional expansions, gains or losses beside those depicted in the figure. Note that LECA cyclin G/I likely gave rise to plant cyclin D and share a common ancestor to the metazoan cyclins D and G/I. Gain of other cyclin families are depicted as regular letters at different stems. (TIF) [file pgen.1008584.s015.tif]
