# Supplementary material for: Cell cycle transcriptomics of Capsaspora provides insights into the evolution of cyclin-CDK machinery
Source: PLoS Genet. 2020 Mar 16;16(3):e1008584. doi: 10.1371/journal.pgen.1008584 (PMC7098662; doi:10.1371/journal.pgen.1008584)
Supplement: Attachment — Submitted filename: 20191128_REVIEWERS_COMMENTS.docx [file pgen.1008584.s036.docx]

Dear Joseph Heitman and Bret Payseur,

Thank you very much for reviewing our manuscript entitled “*Gradual evolution of cell cycle regulation by cyclin-dependent kinases during the transition to animal multicellularity*” (manuscript PGENETICS-D-19-01130). We are pleased that all reviewers found our work interesting and we are grateful for the comments and insightful suggestions that would improve both the layout and the understanding of the results presented.

We have made efforts to address all concerns. We have performed extra experiments (qPCRs), changed figures and text, modified the title (which now reads as “*Cell cycle transcriptomics of Capsaspora provides insights into the evolution of cyclin-CDK machinery*”), and tone down some of the evolutionary speculation.

The following is a point-by-point response to the reviewers’ comments. We hope you will find our changes satisfactory.

Thank you for your consideration of this manuscript.

Sincerely yours,

Iñaki Ruiz-Trillo

Reviewer #1: Synopsis: There is a growing interest in the cell biology of early-diverging holozoans (Monosiga, Salpingoeca, Sphaeroforma, and Capsaspora) because they provide insights on the early ancestor of animals. Here, the authors used hydroxyurea to block the cell cycle of Capsaspora and then synchronously release the population upon wash out (Fig. 1). They used microscopy to analyze cell division, tubulin staining, DNA straining of synchronized cells (Fig. 2) to quantify the synchrony and timing of the cell cycle (mitosis is 10.5 hours after release, total cell cycle period is ~12 hours). The authors then used RNA-seq to identify ~800 cell cycle regulated genes (10% of the genome) after synchronous block-release; Figs. 3-4. This dataset forms the basis of their bioinformatic analysis. The main claims are that Capsaspora cyclins E, A, B have similar timing to their animal counterparts (Fig. 5) and Capsaspora cell cycle transcription program resembles humans more than yeasts and plant (Fig. 6). Knowing the timing of cyclin and CDK gene expression in early-diverging holozoa is important for understanding the principles of cell cycle regulation and its conservation across eukaryotes.

Strengths: This work is novel because it is (to my knowledge) the first cell cycle expression dataset in an early-diverging holozoan. If so, the authors should state this more explicitly and put their work in context. I think the manuscript will be of broad interest to the cell cycle and evolutionary genomics community. The authors worked with a non-model organism and made great use of genomics, which should inspire other groups working with early-diverging holozoa or non-model organisms.

Weaknesses: The analysis of RNA-seq replicates is well done. The density of time sampling was high (45 minutes for 12 hours). The use of two methods for identifying periodic genes with 11 to 13-hour period (already known from Fig. 2) is good, and the circular structure in the two principle components (Fig. 3D) is reassuring. However, I could not find experiments that validate the timing of Cyclin E, A, B and Cdk1 expression observed in RNA-seq experiments, e.g. Fig. 5. The authors also make some claims regarding the expansion of cyclins and CDKs that are inaccurate and, thus, should be rewritten; see Comments below. Both of these weaknesses are relatively straightforward to address.

Major comments:

** Lines 197-201 and Discussion: Although it is exciting to see a mitotic spindle in the absence of a canonical MTOC, the absence of gamma-tubulin ring complex genes (i.e. evidence for lack of MTOC) could also be due to poor annotation or an incomplete genome? How confident are the authors that Capsaspora genome does not encode for canonical MTOC? Are Sphaeroforma, Monosiga, and Salpingoeca genomes also missing these components? If so, this would suggest that these genes are missing. Please elaborate and rewrite.

***Thanks. To address this, we performed BLAST best reciprocal hit of several human core centrosomal proteins as well as yeast spindle-pole body (SPB) proteins in the proteomes of multiple unicellular holozoan lineages. The results, included as Fig. S2, clearly emphasize the absence of several proteins involved in the canonical MTOC as well as absence of SPB proteins. It is worth mentioning that the genome of Capsaspora was done by Sanger sequencing and it is a very complete and well annotated genome, which minimizes the chances of lacking genes because of poor annotation or incomplete genome. We have clarified all these results, including the fact that *Capsaspora* indeed has gamma-tubulin. Materials and methods have also been updated accordingly.**

** One of the main conclusions is the conservation of gene expression timing of Cyclin E, A, B and CDK expression between Capsaspora and humans. However, I could not find experiments to validate the timing of Cyclin E, A, B and CDK expression observed in RNA-seq experiments. At a minimum, some alternative method to validate transcript levels (RT-qPCR, Northern blot) is needed. More ambitiously, one could measure Cyclin E, A, B protein levels using Western blot. This would really emphasize similarities between Capsaspora and humans, and improve significance and impact.

***We do agree that a Western-blot of all these key regulators would have been ideal. However, this would require tailor-made antibodies and we believe that this is beyond the scope of this study. However, in order to address the reviewer concern we performed qPCR experiments for cyclins B, E, and CDK1/2/3, obtained from two novel HU-block and release experiments independent from the original transcriptomic analysis. The results were consistent with the RNAseq results shown in Fig. 5. We have included the results of the RT-qPCR in Fig S11C and updated the results and the methods accordingly.**

** According to the phylogenetic tree, Capsaspora lacks a Cyclin D, but it has a Cyclin F and two Cyclins G (which cluster with Cyclin E). Have the authors analyzed the expression profile of these cyclins in Capsaspora compared to humans? This would improve significance and impact.

***We have plotted the expression profile of the Capsaspora co-ortholog of cyclin G/I (CAOG_00179) in Fig. S11. As for cyclin F, we assume the reviewer referred to the gene CAOG_01199, which in the phylogeny can be mistaken as stemming from the cyclin F clade when it is an unclassified cyclin. This cyclin seems to be expressed at the onset of G1, and has been added to the plot alongside cyclin G/I. We have added these cyclins to the main text, linking the role of Cyclin G as a mitotic repressor with the timing of expression in *Capsaspora*.**

** Ref. 2 has a Cyclin and CDK phylogeny (Figure 2-Figsupp2, Figsupp6) with a broad number of eukaryotic outgroups (including Capsaspora, Salpingoeca, Sphaeroforma, Monosiga). This reference will be helpful in two ways:

(i) Lines 318-332: The CDK phylogeny of Ref. 2 places holozoa CDK1/2/3 with "CDKA" (the ancestral CDK1/2/3 found in plants and deep protists). This provides better evidence in support of scenario 2 (CDK1 and CDK2/3 are metazoan-specific gene duplications).

***Done**

(ii) Lines 482-486: LECA already had one CDK and several Cyclins (A, B, D/E/F/G/etc..). The evolutionary intermediate state (of multiple cyclins) is found in many other eukaryotes, not just Capsaspora; see Ref. 2. The sub-functionalization of cyclins happened between FECA (first common ancestor) and LECA. The authors should rewrite their discussion accordingly.

***We agree with this reference in that there likely was a diversified toolkit of cyclin A,B, and D/E/F/G cyclins prior to the last eukaryotic common ancestor. Based on our analysis, some of these diversifications might have occurred between LECA and the last common ancestor of animals. For example, metazoan cyclins D, G and I on Fig. S8 do not group as sister groups -such a thing would imply a metazoan-specific duplication- but are more phylogenetically related mainly with unicellular Holozoa, suggesting that these duplications occurred in an ancestor prior to Holozoa. Likewise, plant cyclins D are more phylogenetically related to metazoan cyclins G/I both in Ref. #2 and in our analysis, suggesting that the diversification of metazoan cyclin D occurred, precisely, at the onset of animals.**

**According to Ref no. 2 and our data, we have edited and expanded the discussion of evolution of cyclins in eukaryotes, and edited Figure 7 to reflect such arguments. We have also included for clarity S15 Fig, where the different expansions and divergences of cyclin/CDK families are shown.**

Minor comments:

** Line 187: I find this comparison awkward and meaningless (especially when comparing to yeast).

***We have removed this comparison.**

** Why is the JTK Rank clustered into vertical bands in Figure 3B? Please clarify.

***This has to do with the fact that JTK ranks multiple genes with the same rank (i.e., produces many tied ranks), resulting in such clustering. This can also be observed in other figures such as Fig. S14.**

** Line 254, Fig. 4: Are cells that are blocked in DNA replication due to hydroxyurea and then released still in G1/S? I ask because the GO enrichment categories are more consistent with S phase. Perhaps you could clarify in the main text how you arrived at these classifications. Is it based on DNA content and cell cycle events in Figs. 1-2 and/or GO categories? Perhaps label the inferred phases in Figs. 1-2?

***We consider those cells to be in G1/S based on the DNA content of the first time point (Fig. 1C). Despite the results of the GO enrichment analysis, we decided to name the clusters accordingly to the DNA content observed in Figure 1C, which was our initial observation. We have labelled the different time points of Fig. 1C and the hour axis of Fig. 2C.**

** Line 297: "Cdc2" (old name, based on homology to Pombe) should be "CDK1"? The authors use CDK1,2,3,4,etc.. throughout their manuscript.

***We agree. We have changed the name to CDK1.**

** Line 375: Could the authors add a sentence to describe how they calculate "gene age", i.e. gene found only in Capsaspora is youngest, gene found in all species is oldest?

***Sure. We have expanded the sentence, now explaining how we calculate gene age and with a reference to our Methods section.**

** Line 419: Can you elaborate on the core set of genes of conserved oscillatory expression? This is novel and interesting.

***We have elaborated on this. This set comprises fifty-five of our groups of orthologs that are periodic in all the species. For each species independently, we did GO enrichment analyses of those orthogroups with periodic genes in all species respect to the total orthogroups -periodic or not- present in all species. Many of these enriched functions are shared across datasets of the five species, such as protein phosphorylation, DNA-protein binding, and different GO terms related to chromatin dynamics. We have also included a list of the putative identities (as by eggnog annotation) of the *Capsaspora* genes belonging to those orthogroups, where it can be seen that a number of them are related to known cell cycle regulators.**

**We have added a new supplementary figure, S12Fig, where all the enriched GO terms for the five species can be found. We have also updated the main text and the methods to reflect these changes.**

** Line 1144: "Seven" should be “five".

***We have corrected this.**

Reviewer #2: The manuscript by Perez-Posada and colleagues describes the transcriptional regulation of the cell cycle in the unicellular protist Capsaspora and compares its cell cycle-related gene content (especially the CDK-cyclin part) between Capsaspora, two yeasts (Saccharomyces cerevisiae and Schizosaccharomyces pombe) and humans. The manuscript is clearly written and the results are well described. I am not an expert in the molecular biology of the cell cycle, but from an outsider's perspective it appears that the ability to obtain synchronized Capsaspora cell cultures and study the molecular biology of the cell cycle seems a considerable step toward studying the evolution of the cell cycle in animals and their relatives, but also more broadly in eukaryotes.

My main issue with the manuscript is the simplistic evolutionary comparisons and interpretations. I simply don't buy on the basis of the evidence presented the authors' evolutionary inference that the Capsaspora cyclin-CDK system "could represent an intermediate state in the evolution of animal-like cyclin-CDK regulation". Although that may end up being the case, my issue is that the authors' analyses haven't shown this. This is so because all their inferences are based on comparisons between Capsaspora and humans. But as the authors well know, there are many more animals than humans (what about Drosophila, non-vertebrate chordates or non-bilaterians?) and many more protists than Capsaspora (choanoflagellates, Ministeria, etc.). Similarly, there are many more outgroups than just fungi (the authors briefly mention Dictyostelium and plants) that would be very informative.

To address this issue, i believe the authors should greatly expand their search of CDK-cyclin genes in a broader sample of representative eukaryotes and add a figure that summarizes the distribution of these genes in these organisms.

***We thank the reviewer for raising these issues. We now refrain from the way we previously conveyed the message and have indeed edited the evolutionary comparisons in the manuscript, including a more detailed explanation of scenarios in other organisms, and edited figure 7. We have also added a new supplementary, Fig. S15, to reflect a better described scenario of the evolution of cyclins and CDKs found in animals. We further modified the title, results and discussion, in order to better convey the main results of this study.**

Minor comments:

L26: "are present in all eukaryotes" -> "are widely / highly conserved in eukaryotes”

***We have changed this.**

L31: "in a more representative opisthokont" - what is a "representative" opisthokont is highly subjective

***We have changed the phrase to “For a better understanding of the evolution of the cell cycle regulation in opisthokonts,[…]”**

L42-43: "could represent an intermediate state" - how can you distinguish this hypothesis from secondary simplification in Capsaspora?

***We have changed this accordingly; see above.**

L55: "yeasts" -> “fungi"

***Changed**

L140-141: This sentence should be stated as a hypothesis and not as an inference

*****

**Again, see above.**

L229-231: Can you rephrase? not sure i understand what you mean here

***We have rephrased to make clear that the observed correlations mean that the two replicates are very consistent with each other.**

L326-327: how do you find this hypothesis to be "more parsimonious"?

***We find this hypothesis more parsimonious in the sense that it does not require any gene loss, whereas the other involves independent losses of an hypothetical paralog in all the holozoan lineages except Metazoa. We have expanded the main text to reflect our reasoning.**

L341: "ancient" -> "conserved" (the genes present in the Capsaspora genome are modern but conserved, they are not ancient)

***Changed**

L465-467: Which study was this? Please include a citation. And how much more limited was their sampling? All you've added is Capsaspora, which may or may not turn out to be a good model

***We have added a citation to the study of Jensen et al, 2006.**

Figure 7: i would definitely add more representative eukaryotic species here

***According to the discussion, we have edited figure 7 to include the ancestral scenario, and further elaborated these points in the text. Likewise, Fig S15 provides more detail into the evolution of the cyclin-CDK content found in animals in the context of eukaryotic evolution.**

Reviewer #3: The manuscript titled “Gradual evolution of cell cycle regulation by cyclin-dependent kinases during the transition to animal multicellularity” provides useful and important insights into the diversity and evolution of cell cycle control within the opisthokonts. The strength of the work lies in the development of a clean and reversible cell cycle synchronization protocol for vegtative Capsaspora cultures. The authors have capitalized on this achievement to identify cyclically regulated genes. They then go on to repurpose their analysis pipeline to identify cyclically expressed genes using published yeast, and both human Arabidopsis cell culture experiments to explore overlap in genes that are cyclically expressed.

Although the work presented in this manuscript is correlative, the experiments and analyses are appropriate first steps given how little is known about the biology of the system. Obvious next steps will be to prove that cyclical expression is dependent on transcription, identify transcriptional regulators (the transcription factor binding sites and proteins they bind), and/or show that the proposed regulators are actually controlling the cell cycle. Although it is tempting to ask for these analyses in the present paper, each item in the above list would require months (or years) of work to in a non-model system, are unnecessary to support the core claims made by the authors, and would drastically alter the scope of the paper and therefore necessitate multiple rounds of peer review.

***We thank the reviewer for appreciating we are dealing with a non-model system.**

The following points, however, should be addressed to ensure that the claims made in the paper are supported by the provided evidence, as well as to enhance the value of the reported data through discussion and comparison of existing literature.

Major points:

Major Point 1: In my opinion, the title is unnecessarily hyperbolic, and echos the oversimplification depicted in Figure 7 that depicts Capsaspora as an intermediate between human cells and yeast (which has been, as the authors describe, shown to be divergent relative to other fungi and protists). The data in the paper does indicate that aspects of human cell cycle regulation were already in place before the last common ancestor of animals. However, to extend that finding to claim that there was a gradual accumulation of cell cycle complexity associated with the evolution of animal multicellularity requires analysis of multiple animal lineages (Drosophila might be one to look at, among others) and additional non-opisthokonts are needed (Dictyostelium comes to mind). This may be possible to do by adding a paragraph discussion section that compares the new analyses to previous analyses of cell cycle regulation in additional lineages. If this is not feasible, the text and title should be edited to align with the findings of the paper.

***We agree. We have changed the title and expanded the discussion to clarify the points raised.**

Major Point 2: The sequencing and cytometry results are based on two replicate experiments. Given the number of timepoints involved, 3 biological replicates (as is customary in the field) may not have been feasible. However, this makes it even more important to describe in detail how distinct the two replicates are. The methods as described beginning on Line 522 suggest a scenario wherein a culture was split into 2 flasks, and were later treated treated in parallel. If this is correct, it should be stated as such. Furthermore, it may be more accurate to call these technical replicates as sister flasks may not display the biological variability needed for biological replicates (biological replicates should, if at all possible, be separated in time to ensure robust data).

***We have clarified in the text that the samples were collected at the same time but come from two independently propagated cell lines, with a minimum of two transfers before the experiment. This issue was taken into account for the experimental design of the RT-qPCRs suggested by Reviewer no.1, and the cultures were propagated independently for several transfers as well. We observed the same trend between the RNA-seq and the RT-qPCRs, two experiments separated in time, for which we are confident on the robustness of the replicates.**

Major Point 3: The decision to select genes for further analysis by ranking of P-values and then selection of a specific number genes should be justified (in contrast to analyzing all genes that meet a certain threshold p-value). For each analysis where this approach was taken, the maximum p-values included for the analysis should be reported, including the appropriate multiple-testing correction.

***Since we have summed two ranks coming from different methods and different p-values, assigning a final p-value is difficult. Indeed, using a cutoff based on a number of genes rather than threshold p-value is customary in the field, as previously done by different authors (such as Spellman et al., 1998, Whitfield et al., 2002; Orlando et al., 2008; Kelliher et al., 2016 )(all included in the manuscript).**

Major Point 4: Because this organism is diverged from model organisms, the behavior of *Capsaspora* cells after release from HU should be compared to what happens in other organisms. Is the 2 hour lag from wash out of HU typical (normalizing for total cell cycle time)? If not, what might be going on?

***We chose to start sampling after 2 hours because we reasoned that, immediately after release from arrest, the transcriptional profile would be dominated by stress response genes. This is also commonly done in yeast synchronization experiments (see for example the Orlando et al paper cited in our manuscript, where the first time point is sampled at around 30 minutes after synchronization). As for why S-phase is delayed for 3 hours after release from the HU block, we don't have an answer, but it likely lies in the fact that the cells have to adapt to the release from arrest and resume the cell cycle. Indeed, in fission yeast -with a cell cycle of four hours-, a delay of 30 minutes between release from the HU block and S phase was observed (Xu et al., Genetics 2016), and in budding yeast, it takes between forty to sixty minutes to get into S phase (Su et al., Mol Cel Biol 2005), the total cycle lasting for 100 minutes.**

Major Point 5: The method section for synchronization does not have enough detail to replicate the experiments. How many times were cells washed? With what volume of media? It may be useful to others in this and related fields to include a step-by-step protocol as a supplemental document.

***We have expanded the experimental procedures in the Methods section, now including a step-wise protocol.**

Minor Points:

Minor Point 1: In the abstract, it is stated that Capsaspora is “more representative” of opisthokonts. Without a large sample size across a large diversity of lineages, it is impossible to say whether a particular phenotype or genotype is “more representative”. Most likely, the authors simply mean that we need to broaden our perspective (which is clearly true and an important point) and can clarify the language here.

***We have changed the phrase to “For a better understanding of the evolution of the cell cycle regulation in opisthokonts, […]”.**

Minor Point 2: In the initial description of cell cycle synchronization (paragraph that begins on Line 159), it would be useful to state the doubling time of untreated adherent Capsaspora cells. This would provide context with which to evaluate the timing of cell cycle synchronization with hydroxyurea. If this number is unknown, it should be calculated and reported here.

***We have measured the doubling time of non-synchronized cultures of *Capsaspora*, using Optical Density (OD=600nm) in a time-course lasting 30 hours. The doubling time of non-synchronized cultures is approximately 8.5-9 hours (S1 Fig), whereas the doubling time from the synchronized cultures in Fig. 1C is 9 to 10 hours. Despite the slight difference, probably due to the treatment with Hydroxyurea, we believe our data encompasses a full round of the cell cycle fairly similar to what happens in non-treated cells. We have referenced this information in the main text.**

Minor Point 3: In line 198, it states that it has been previously reported that Capsaspora has no gamma-tubulin ring complex, and cites a review on the evolution of MTOCs. I was not able to find reference to a lack of gamma-tubulin ring complexes in Capsaspora in the indicated paper (Figure 1 of the cited paper indicates Capsaspora does not have a SPD-2/CEP192 homolog, but this is not part of the gamma tubulin ring complex). Moreover, lack of Gamma tubulin and GCP2, 3, 4, 5, and 6 would be very surprising as this complex typically is used to nucleate microtubules in species whether or not they have MTOCs. But, more to the point, the presence or absence of the gamma-turc complex is a separate question from whether or not cells display centrosomes. The authors should clarify exactly what they mean here and ensure that the citations are what they intend.

***We have previously addressed a similar comment from Reviewer #1, please see above.**

Minor Point 4: The manuscript would benefit from copy editing for clarity. In particular, the sentence beginning on Line 344 is very confusing.

***We have edited this sentence, hoping the writing is clearer now.**

Minor Point 5: The cells in the panels of Fig. 2A are too small. Over half of each image could be cropped, and the remaining portion enlarged to allow the reader to see the dividing cell. Also, a time point before the cells round up should be included. Finally, the most common term is alpha-tubulin (not tubulin-alpha).

***We have cropped the figures in Fig.2A, as suggested. We have also added an arrowhead showing the same cell dividing. We clarify here that time zero in Fig. 2A corresponds to cells pre-rounding up, as can be observed by the non-round shape and the presence of filopodia in that same cell.**

Minor Point 6: Fig. 2C axis units should be %age of cells in mitosis (the methods indicate that 100 cells were analyzed per time point).

***We clarify here that we analyzed a total of 101 cells in the whole experiment comprising figures 2C-D. Fig. 2C depicts the distribution in time of the 101 mitotic cells that were measured, and Fig. 2D depicts the relative cell area of each pair of daughter cells. We have edited the text to make our analysis understandable, as well as the methods.**

Minor Point 7: Fig. 6: A, right panel AND Fig 6 B: These charts are not intuitive, nor are they well described in the figure legend. These panel need to be redesigned to be understandable and/or the figure legend written to walk the reader step-by-step through what is contained in the panel.

***We have edited the figure by adding titles, and have expanded the figure legend to guide the reader, hopefully making it more understandable.**

Minor Point 8: Fig 6C: the three colors are too close in hue to be easily discerned.

***We have edited the colors.**

Reviewer #4: PGENETICS-D-19-01130 Perez-Posada et al.

The authors present a beautiful cell-cycle analysis of the unicellular, non-model organism Capsaspora owczarzaki, a species related to animal cells. They developed a synchrony/release time-series protocol using HU as a synchronization agent and analyzed cell-cycle progression by microscopy, flow cytometry, and RNA-seq. The punchline of the study from the abstract, summary and text appears to be that C. owczarzaki is likely an intermediate between yeasts and animal cells where orthologs of cyclins A, E, and B are binding to a single CDK. As well, the authors suggest that the cell cycle transcriptional program in C. owczarzaki is more similar to humans than yeasts.

The data presented are beautiful and are a very welcome addition to the burgeoning collection of high quality cell-cycle-time-series data in the community. However, a punchline that C. owczarzaki is an intermediate species with cyclin A, B, and E orthologs but only one CDK is a bit confusing, as that evidence comes largely from genome sequence data, and not the transcriptome (or microscopy/cytometry) data presented. Expansion of the CDK genes in animal cell lineages could also be observed directly from genome sequence. Thus, the major conclusion stemming from the transcriptome data analysis seems to be that the cell-cycle transcriptional program is more similar to humans than yeast. Comparing these programs is an important and difficult task as the underlying gene regulatory networks controlling the program in yeast has been suggested to function as the central cell-cycle oscillator. As presented, the conclusion that the program is more similar to humans is not obvious and needs to be specified further. As well, it is not clear that the conclusion is robust to the analytical methods, so some sensitivity testing should be done.

***Thanks!**

Specific comments:

1) On line 422 the authors state; “Overall, our cross-species comparison of the periodic gene expression programs revealed that the Capsaspora periodic gene expression program is more similar to human cells that to current unicellular model systems for the cell cycle…”. It is not clear what the authors mean by “more similar”. What precisely is the similarity metric they are using? Number of orthologs? Number of periodic orthologs? Ordering of orthologous genes? Number of genes in each cluster?

***We have clarified that by similarity we mean number of periodic orthologs.**

There are numerous comparisons one could make, especially in higher dimensions. These metrics need to be specified. Any metric that involves making calls on orthologs or making calls on periodic gene lists may be sensitive to the choices made by the methods. Thus, care needs to be made to test the sensitivity of these calls (See below).

The authors reference the work of Buchler, Skotheim, and Cross, who discovered that although the sequences of the genes making up the gene regulatory network controlling cell cycle transcription are unrelated in yeast and humans, the network topology is identical. This finding should be discussed as it suggests an additional way to measure evolutionary similarity beyond sequence homology.

***We appreciate the importance of this. However, as we were not able to perform any functional tests on genes of interest, any thoughts or insights concerning network topology in *Capsaspora* would be pure speculation at this moment.**

2) There is no clear or obvious way to define cell-cycle regulated genes. There are many algorithmic methods that produce rank-ordered lists using a variety of definitions of “periodic”. The tricky part is always setting a sensible cut-off because these lists tend not to be bimodal (cell-cycle-regulated and not cell-cycle-regulated). The authors set a cut-off for their comparisons at 10% of the total genome, and it seems like this could influence their measure of periodic orthology. For the S. cerevisiae, the estimates of multiple studies suggest that ~ 20% of the genome appears to be under substantial cell cycle control. It makes sense that a single cell organism may have a greater part of its genome dedicated to cell division. The authors should broaden that cut-off and once the define similarity metrics precisely, they can ask whether their conclusions are robust to the choice of the size of the periodic gene sets they are querying.

***We thank the reviewer for such suggestion. We performed prior analyses relaxing or constraining the cutoff, and have included a supplementary figure (S13 Fig) with a clustering, Gene Ontology enrichment, and pairwise-species orthogroup comparisons for a dataset of 1600 periodic genes in *Capsaspora*. Briefly, we observed a similar trend as previously reported in Figures 3, 4, and 6.**

3) Although there is no right or wrong way to define a periodic gene set, the method used by the authors is somewhat confusing. JTK cycle requires the user to input an expected period length or range of period lengths. Although I have no experience with RAIN, it appears it has a similar requirement. In Figures 1, 3, and 4 the authors make the case for the fact that in the 13.25-hour time-series experiment, they see cells return to the initial G1 (G1/S) state (By the way, the flow cytometry data says 13.25 in figure1 and Figure 4a the RNA-seq data says 13.15). The return to G1 can be observed in the flow cytometry data (Fig. 1) and the RNA-seq data (Fig. 4), arguing the cell-cycle period is in the 13-hour range. Yet, in the JTK analysis, the authors used a range of periods from 14 to 16 hours, and for the RAIN algorithm, they used a period of 16 hours. The authors need to justify these choices as they do not fit the data presented. Again, they should demonstrate that their choice of periods does not influence their final conclusions.

***We have used the period as 14-16 data points, which corresponds to 14-16 (JTK-CYCLE) and 16 (RAIN) time points, not hours. As time points were sampled every 45 minutes, this indeed corresponds to the actual cell cycle period based on experiments. We have further clarified this in the methods section. We have also edited the label in Fig. 4A to 13.25.**

4) I don’t have the expertise to evaluate their methods for making calls on orthology, but it also seems like a complex and tricky business that could substantially influence the conclusions.

***Indeed. This is why we did two analyses, the orthogroup analyses, which has the advantage of finding orthologs of a large fraction of genes, and the phylome analysis based on the published data, which only reports 1:1 orthologs, and thus omits many genes, but is more accurate. Both analyses are reported in our manuscript, and the general conclusions hold regardless on the method used for making calls on orthology.**

5) There is growing evidence that evolutionary distance or mechanism can be determined simply by orthology is short-sighted. Given the findings of Buchler, Skotheim, and Cross on the evolution of cell cycle transcription in humans and fungal lineages, it would be nice to see the work presented here in a broader context. Are there E2F/SBF/MBF and Rb/Whi5 orthologs? Any evidence they are controlling G1/S transcription or acting in similar network motifs?

***Capsaspora has E2F and Rb, but not MBF/SBF system. We now include the transcriptional profile of these regulators in the manuscript and in Figure 5C. In this same figure, we also include the profile of E2F7-another E2F factor known to act as a repressor in mammalian cells, which is periodic in *Capsaspora*. As for what might be the targets of Rb/E2F in *Capsaspora*, further experimental work is required that we unfortunately believe is beyond the scope of the paper.**

Small comments:

On line 225, the authors state; “ we confirmed that top-ranked genes showed oscillatory behavior”. How exactly was oscillatory behavior confirmed? I’m guessing it was visual inspection? The eyeball test is certainly a legitimate method for checking, but whatever the method is should be specified.

***We have clarified that it was visual inspection of the expression profile.**

For Figure 4A, the authors show normalized expression values. From the description in the methods I think what they are presenting is a Z-score. If it is, it should be labeled as such in the figure. If it’s not, then some rationale for the normalization method should be stated.

***We have clarified that we meant Z-score and edited the y axes in the plots.**
